# Supplementary figures and images for: Genome-Wide Identification and Expression Analysis of GASA Genes in Hevea brasiliensis Reveals Their Involvement in Response to Cold Stress
Source: Int J Mol Sci. 2025 Apr 7;26(7):3454. doi: 10.3390/ijms26073454 (PMC11990028; doi:10.3390/ijms26073454)

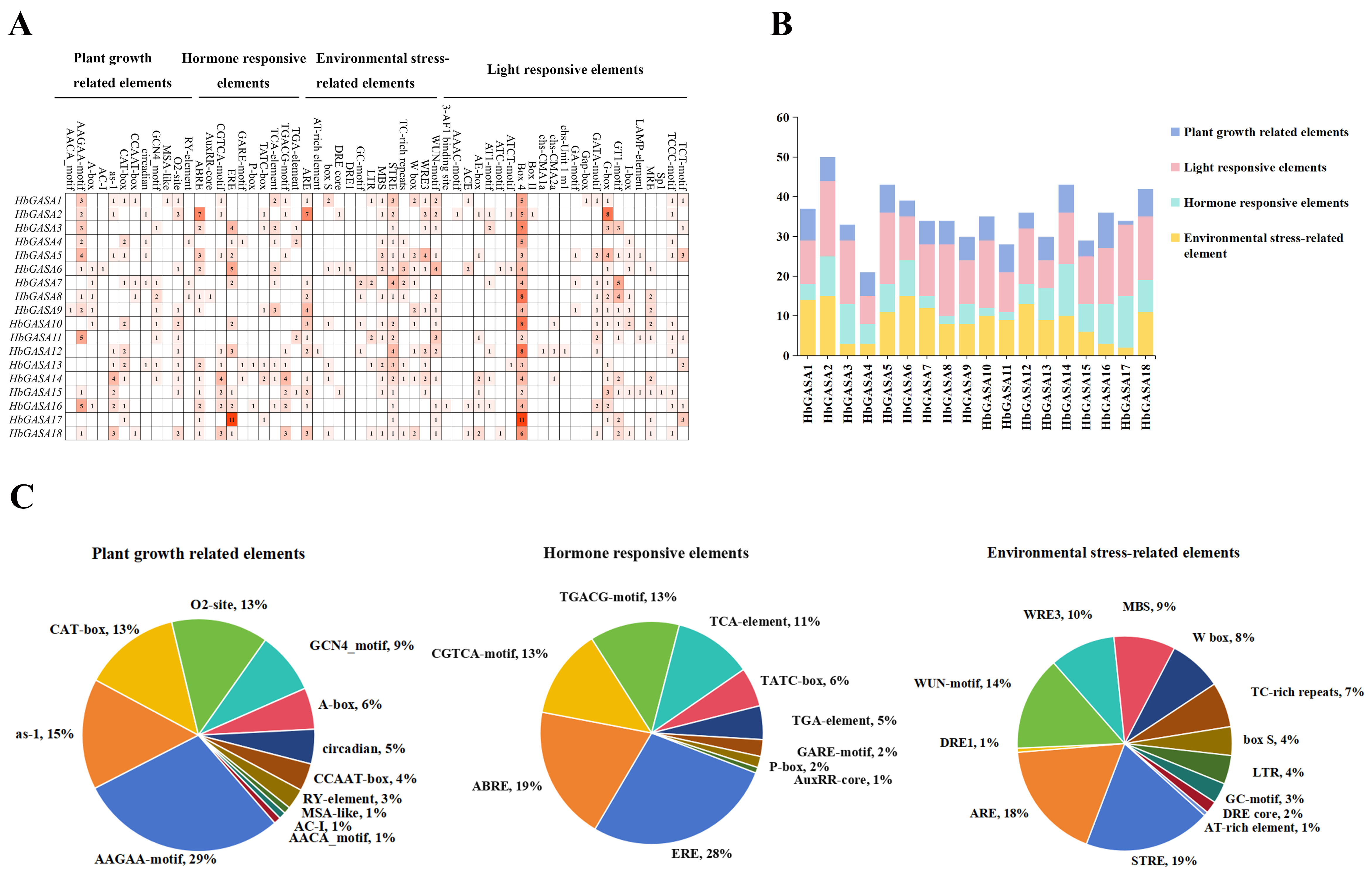

Supplement: Supplementary file 1 [file ijms-26-03454-s001.zip › Figure.S1.png]
